# Supplementary material for: Assessment of the Impact of a Meningitis/Encephalitis Panel on Hospital Length of Stay: A Systematic Review and Meta-Analysis
Source: Antibiotics (Basel). 2022 Jul 30;11(8):1028. doi: 10.3390/antibiotics11081028 (PMC9405449; doi:10.3390/antibiotics11081028)
Supplement: Supplementary file 1 [file antibiotics-11-01028-s001.zip › antibiotics-1781724-supplementary.pdf]

SUPPLEMENTARY MATERIALS

Assessment of the Impact of a Meningitis/Encephalitis Panel on Hospital Length of Stay: A Systematic Review and Meta-Analysis

Kyle D. Hueth[1], Philippe Thompson-Leduc[2], Todor I. Totev[3], Katherine Milbers[2], Tristan T. Timbrook[1], Noam Kirson[3], Rodrigo Hasbun[4]

[1] BioFire Diagnostics, LLC, 515 Colorow Rd, Salt Lake City, UT 84108, USA
[2] Analysis Group, Inc., Deloitte Tower, 1190 Av. des Canadiens-de-Montréal Suite 1500, Montreal, Quebec H3B 0G7, Canada
[3] Analysis Group, Inc., 111 Huntington Ave 14th floor, Boston, MA 02199, USA
[4] McGovern Medical School, University of Texas Health Sciences Center at Houston, 6410 Fannin St #1014, Houston, TX 77030, USA

Corresponding Author:

Philippe Thompson-Leduc
Manager
Analysis Group, Inc.
Deloitte Tower, 1190 Avenue des Canadiens-de-Montréal Suite 1500
Montréal, Quebec H3B 0G7
Canada
Tel: 514-871-4238
Email: Philippe.Thompson-Leduc@analysisgroup.com

Supplementary Table S1. PRISMA 2020 Checklist

Section and Topic	Item #	Checklist item	Location where item is reported
**TITLE**			
Title	1	Identify the report as a systematic review.	Title page
**ABSTRACT**			
Abstract	2	See the PRISMA 2020 for Abstracts checklist.	See below
**INTRODUCTION**			
Rationale	3	Describe the rationale for the review in the context of existing knowledge.	Sec. 1, p.1-2
Objectives	4	Provide an explicit statement of the objective(s) or question(s) the review addresses.	Sec. 1.2, p. 2
**METHODS**			
Eligibility criteria	5	Specify the inclusion and exclusion criteria for the review and how studies were grouped for the syntheses.	Sec. 2.1, p. 2-3
Information sources	6	Specify all databases, registers, websites, organisations, reference lists and other sources searched or consulted to identify studies. Specify the date when each source was last searched or consulted.	Sec. 2.1, p. 2-3
Search strategy	7	Present the full search strategies for all databases, registers and websites, including any filters and limits used.	Supp. Table 3
Selection process	8	Specify the methods used to decide whether a study met the inclusion criteria of the review, including how many reviewers screened each record and each report retrieved, whether they worked independently, and if applicable, details of automation tools used in the process.	Sec. 2.1, p. 2-3
Data collection process	9	Specify the methods used to collect data from reports, including how many reviewers collected data from each report, whether they worked independently, any processes for obtaining or confirming data from study investigators, and if applicable, details of automation tools used in the process.	Sec. 2.2, p. 3
Data items	10a	List and define all outcomes for which data were sought. Specify whether all results that were compatible with each outcome domain in each study were sought (e.g. for all measures, time points, analyses), and if not, the methods used to decide which results to collect.	Sec. 2.2, p. 3
	10b	List and define all other variables for which data were sought (e.g. participant and intervention characteristics, funding sources). Describe any assumptions made about any missing or unclear information.	Sec. 2.2, p. 3
Study risk of bias assessment	11	Specify the methods used to assess risk of bias in the included studies, including details of the tool(s) used, how many reviewers assessed each study and whether they worked independently, and if applicable, details of automation tools used in the process.	Sec. 2.4, p. 3-4
Effect measures	12	Specify for each outcome the effect measure(s) (e.g. risk ratio, mean difference) used in the synthesis or presentation of results.	Sec. 2.2, p. 3
Synthesis methods	13a	Describe the processes used to decide which studies were eligible for each synthesis (e.g. tabulating the study intervention characteristics and comparing against the planned groups for each synthesis (item #5)).	Sec. 2.2, p. 3
	13b	Describe any methods required to prepare the data for presentation or synthesis, such as handling of missing summary statistics, or data conversions.	Sec. 2.2, p. 3
	13c	Describe any methods used to tabulate or visually display results of individual studies and syntheses.	Sec. 2.2, p. 3
	13d	Describe any methods used to synthesize results and provide a rationale for the choice(s). If meta-analysis was performed, describe the model(s), method(s) to identify the presence and extent of statistical heterogeneity, and software package(s) used.	Sec. 2.2, p. 3
	13e	Describe any methods used to explore possible causes of heterogeneity among study results (e.g. subgroup analysis, meta-regression).	Sec. 2.4, p. 3-4
	13f	Describe any sensitivity analyses conducted to assess robustness of the synthesized results.	Sec. 2.3, p. 3
Reporting bias assessment	14	Describe any methods used to assess risk of bias due to missing results in a synthesis (arising from reporting biases).	N/A
Certainty assessment	15	Describe any methods used to assess certainty (or confidence) in the body of evidence for an outcome.	N/A

Section and Topic	Item #	Checklist item	Location where item is reported
**RESULTS**			
Study selection	16a	Describe the results of the search and selection process, from the number of records identified in the search to the number of studies included in the review, ideally using a flow diagram.	Figure 1
	16b	Cite studies that might appear to meet the inclusion criteria, but which were excluded, and explain why they were excluded.	Sec. 3.1, p. 4
Study characteristics	17	Cite each included study and present its characteristics.	Table 1
Risk of bias in studies	18	Present assessments of risk of bias for each included study.	Supp. Tables 4 & 5
Results of individual studies	19	For all outcomes, present, for each study: (a) summary statistics for each group (where appropriate) and (b) an effect estimate and its precision (e.g. confidence/credible interval), ideally using structured tables or plots.	Figures 2, 3 and 4
Results of syntheses	20a	For each synthesis, briefly summarise the characteristics and risk of bias among contributing studies.	Sec. 3.2-3.4, p. 7-8
	20b	Present results of all statistical syntheses conducted. If meta-analysis was done, present for each the summary estimate and its precision (e.g. confidence/credible interval) and measures of statistical heterogeneity. If comparing groups, describe the direction of the effect.	Figures 2, 3 and 4
	20c	Present results of all investigations of possible causes of heterogeneity among study results.	Sec. 3.2-3.4, p. 7-8 and Sec. 4, p. 9
	20d	Present results of all sensitivity analyses conducted to assess the robustness of the synthesized results.	Sec. 4, p. 9
Reporting biases	21	Present assessments of risk of bias due to missing results (arising from reporting biases) for each synthesis assessed.	N/A
Certainty of evidence	22	Present assessments of certainty (or confidence) in the body of evidence for each outcome assessed.	N/A
**DISCUSSION**			
Discussion	23a	Provide a general interpretation of the results in the context of other evidence.	Sec. 4, p. 9-10
	23b	Discuss any limitations of the evidence included in the review.	Sec. 4, p. 9-10
	23c	Discuss any limitations of the review processes used.	Sec. 4.1, p. 10
	23d	Discuss implications of the results for practice, policy, and future research.	Sec. 5, p. 11
**OTHER INFORMATION**			
Registration and protocol	24a	Provide registration information for the review, including register name and registration number, or state that the review was not registered.	N/A
	24b	Indicate where the review protocol can be accessed, or state that a protocol was not prepared.	N/A
	24c	Describe and explain any amendments to information provided at registration or in the protocol.	N/A
Support	25	Describe sources of financial or non-financial support for the review, and the role of the funders or sponsors in the review.	Funding, p. 11
Competing interests	26	Declare any competing interests of review authors.	Conflicts of Interest. p. 11
Availability of data, code and other materials	27	Report which of the following are publicly available and where they can be found: template data collection forms; data extracted from included studies; data used for all analyses; analytic code; any other materials used in the review.	N/A

From: Page MJ, McKenzie JE, Bossuyt PM, Boutron I, Hoffmann TC, Mulrow CD, et al. The PRISMA 2020 statement: an updated guideline for reporting systematic reviews. BMJ 2021;372:n71. doi: 10.1136/bmj.n71

For more information, visit: http://www.prisma-statement.org/

Supplementary Table S2. PRISMA 2020 for Abstracts Checklist

Section and Topic	Item #	Checklist item	Reported (Yes/No)
**TITLE**			
Title	1	Identify the report as a systematic review.	Yes
**BACKGROUND**			
Objectives	2	Provide an explicit statement of the main objective(s) or question(s) the review addresses.	Yes
**METHODS**			
Eligibility criteria	3	Specify the inclusion and exclusion criteria for the review.	Partially
Information sources	4	Specify the information sources (e.g. databases, registers) used to identify studies and the date when each was last searched.	Partially
Risk of bias	5	Specify the methods used to assess risk of bias in the included studies.	No
Synthesis of results	6	Specify the methods used to present and synthesise results.	Yes
**RESULTS**			
Included studies	7	Give the total number of included studies and participants and summarise relevant characteristics of studies.	Yes
Synthesis of results	8	Present results for main outcomes, preferably indicating the number of included studies and participants for each. If meta-analysis was done, report the summary estimate and confidence/credible interval. If comparing groups, indicate the direction of the effect (i.e. which group is favoured).	Yes
**DISCUSSION**			
Limitations of evidence	9	Provide a brief summary of the limitations of the evidence included in the review (e.g. study risk of bias, inconsistency and imprecision).	No
Interpretation	10	Provide a general interpretation of the results and important implications.	Yes
**OTHER**			
Funding	11	Specify the primary source of funding for the review.	No
Registration	12	Provide the register name and registration number.	N/A

From: Page MJ, McKenzie JE, Bossuyt PM, Boutron I, Hoffmann TC, Mulrow CD, et al. The PRISMA 2020 statement: an updated guideline for reporting systematic reviews. BMJ 2021;372:n71. doi: 10.1136/bmj.n71

For more information, visit: http://www.prisma-statement.org/

Supplementary Table S3. Search Strategy

	Search term	Number of publications
**BioFire FilmArray M/E Panel**		
**1**	biofire or filmarray or "biofire(r)" or "filmarray(r)" or bio fire or film array or "bio fire(r)" or "film array(r)"	1,297
**2**	(meningitis?encephalitis or (meningitis adj2 encephalitis) or meningitis or encephalitis) adj5 panel	314
**3**	(cerebrospinal fluid adj2 (assay* or sample*)) or (cerebro spinal fluid adj2 (assay* or sample*)) or (csf adj2 (assay* or sample*))	19,008
**4**	multiplex adj4 (assay* or panel*)	21,098
**5**	1 or 2 or 3 or 4	41,051
**Meningitis or encephalitis**		
**6**	meningitis or encephalitis or meningoencephalitis or meningitis?encephalitis	277,545
**7**	csf pleocytosis or cerebrospinal fluid pleocytosis or cerebro spinal fluid pleocytosis	2,307
**8**	(central nervous system* adj3 infection*) or (cns adj3 infection*)	27,797
**9**	infecti* workup	897
**10**	6 or 7 or 8 or 9	296,379
**Length of stay**		
**11**	management or managed	4,014,679
**12**	hospitali#ation	699,094
**13**	(duration or "time of" or "length of" or day*) adj4 (stay* or therap* or treatment* or in?patient*)	1,287,207
**14**	hospital* adj4 (day* or discharge*)	380,963
**15**	11 or 12 or 13 or 14	5,713,260
**FA-ME Panel + Length of stay**		
**16**	5 and 10 and 15	833
**Search filters**		
**17**	remove duplicates from 16	587
**18**	limit 17 to yr="2015 -Current"	293
**19**	limit 18 to (autobiography or bibliography or biography or case reports or clinical trial, veterinary or clinical trials, veterinary as topic or clinical trial protocol or clinical trial protocols as topic or comment or congress or consensus development conference or consensus development conference, nih or dataset or dictionary or directory or editorial or "expression of concern" or government publication or guideline or interactive tutorial or interview or lecture or legal case or legislation or letter or news or observational study, veterinary or patient education handout or periodical index or personal narrative or portrait or practice guideline or randomized controlled trial, veterinary or video-audio media or webcast or books or chapter or conference abstract or conference paper or "conference review") [Limit not valid in Ovid MEDLINE(R),Ovid MEDLINE(R) Daily Update,Ovid MEDLINE(R) In-Process,Embase; records were retained]	124
**20**	18 not 19	169

Supplementary Table S4. Risk of Bias 2 Quality Assessments

Author	Domain A	Domain B	Domain C	Domain D	Domain E	Overall risk of bias
Posnakoglou L, 2020	Some concerns	Low risk of bias	Low risk of bias	Low risk of bias	Some concerns	Some concerns

Domains: A) bias arising from the randomization process, B) bias owing to deviations from intended interventions, C) bias owing to missing outcome data, D) bias in measurement of the outcome, E) bias in selection of the reported result. Overall risk of bias evaluated across the five domains.

Source: Sterne JA, Savović J, Page MJ, Elbers RG, et al., RoB 2: a revised tool for assessing risk of bias in randomised trials, BMJ. 2019 Aug 28;366:l4898. doi: 10.1136/bmj.l4898.

Supplementary Table S5. Newcastle-Ottawa Scale Quality Assessments

Author	Selection (max. 4 stars)	Comparability (max. 2 stars)	Outcome (max. 3 stars)	Total Score
Dack K, 2019	****		***	7
Diaz KMO, 2020	****		***	7
DiDiodato G, 2019	****	**	***	9
Evans M, 2020	****		***	7
Hagen A, 2020	****		***	7
McDonald D, 2020	****		***	7
Mina Y, 2019	****		***	7
Moffa MA, 2020	***		***	6
Mostyn A, 2020	***		***	6
Nabower AM, 2019	***	**	***	8
O'Brien MP, 2018	****		***	7
Walker M, 2021	****		***	7

Source: Wells GA, Shea B, O'Connell D, Peterson J, Welch V, Losos M, Tugwell P, The Newcastle-Ottawa Scale (NOS) for assessing the quality of nonrandomised studies in meta-analyses, available online: https://www.ohri.ca/programs/clinical_epidemiology/oxford.asp Accessed 21 July 2022.

Supplementary Figure S1. Hospital Length of Stay (in days) – Stratification by Age

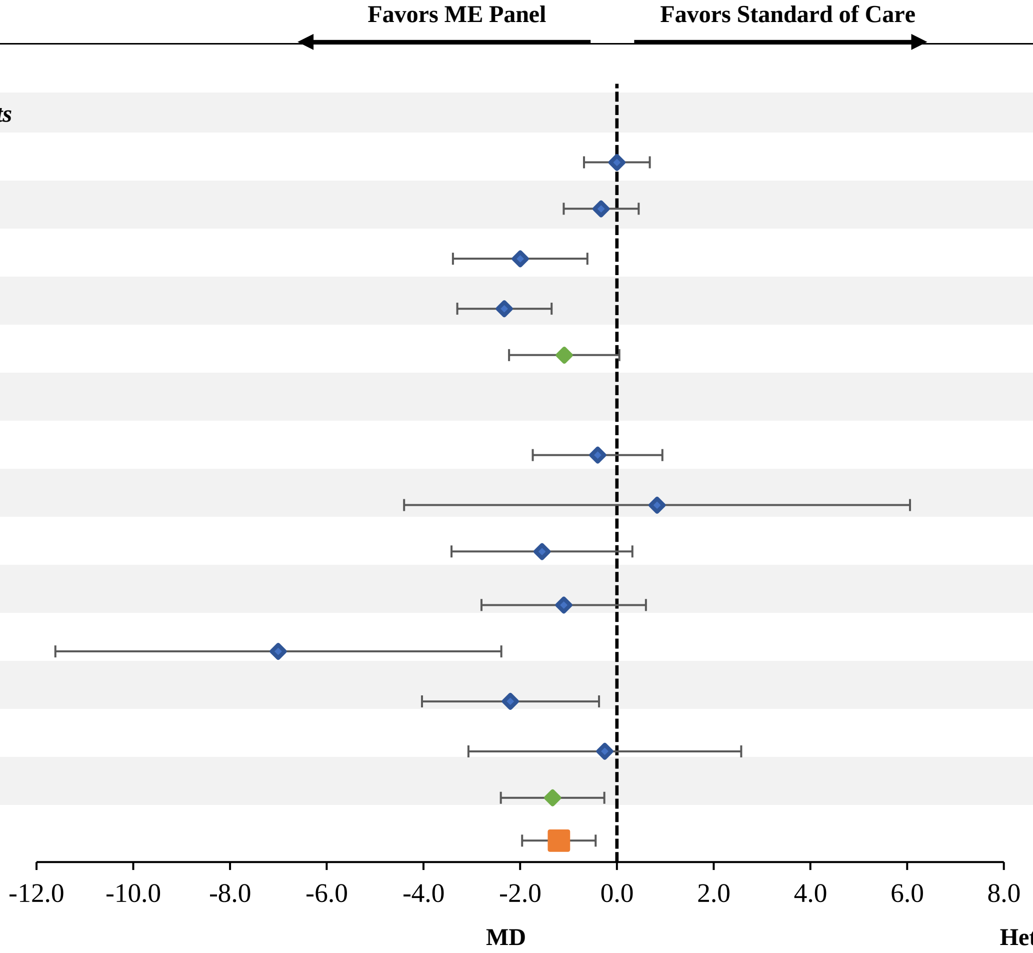

Study	MD (95% CI)	% Weight
Exclusively Pediatric Patients		
Hagen, 2020	0.00 (-0.68, 0.68)	14.92
McDonald, 2020	-0.33 (-1.11, 0.44)	14.40
O'Brien, 2018	-2.00 (-3.39, -0.61)	10.82
Posnakoglou, 2020	-2.33 (-3.31, -1.36)	13.25
**Overall (n= 4)**	**-1.09 (-2.23, 0.05)**	
Other Patients		
Dack, 2019	-0.40 (-1.74, 0.94)	11.11
Diaz, 2020	0.83 (-4.40, 6.06)	1.89
DiDiodato, 2019[a]	-1.55 (-3.42, 0.32)	8.40
Evans, 2020	-1.10 (-2.80, 0.60)	9.18
Mina, 2019	-7.00 (-11.61, -2.39)	2.36
Moffa, 2020	-2.20 (-4.03, -0.37)	8.58
Walker, 2021	-0.25 (-3.07, 2.57)	5.10
**Overall (n= 7)**	**-1.33 (-2.40, -0.26)**	
**Overall, all studies (n= 11)**	**-1.20 (-1.96, -0.44)**	

Heterogeneity, pediatric studies: 84.27%
Heterogeneity, other studies: 38.64%

CI: confidence interval; MD: mean difference; ME: meningitis and/or encephalitis

[a] Analysis was performed on the subgroup of patients whose time to discharge was ≤18 days, n=95.

Supplementary Figure S2. Length of Acyclovir Treatment - Stratification by Age

Study	MD (95% CI)	% Weight
Exclusively Pediatric Patients		
Hagen, 2020	-2.00 (-2.30, -1.70)	18.91
McDonald, 2020	-1.00 (-1.21, -0.79)	19.32
Posnakoglou, 2020	-2.50 (-3.68, -1.32)	11.80
**Overall (n= 3)**	**-1.73 (-2.59, -0.86)**	
Other Patients		
Diaz, 2020	1.17 (-1.01, 3.34)	5.98
Evans, 2020	-0.50 (-0.86, -0.14)	18.59
Moffa, 2020	-2.00 (-2.72, -1.28)	15.74
Walker, 2021[a]	0.67 (-0.80, 2.13)	9.67
**Overall (n= 4)**	**-0.43 (-1.60, 0.73)**	
**Overall (n= 7)**	**-1.14 (-1.78, -0.50)**	

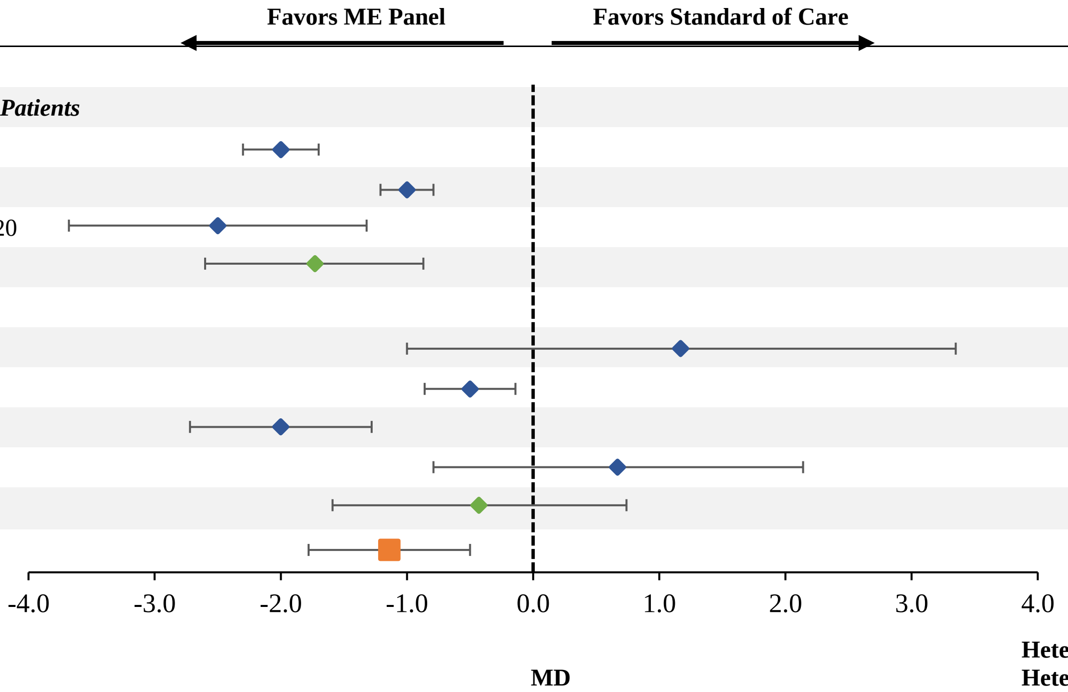

Heterogeneity, pediatric studies: I2 = 93.68%
Heterogeneity, other studies: I2 = 84.84%

CI: confidence interval; MD: mean difference; ME: meningitis and/or encephalitis

[a] One patient (of 19) in the pre-intervention group received an antiviral that was not acyclovir.

Supplementary Figure S3. Days of Treatment with Antibiotics - Stratification by Age

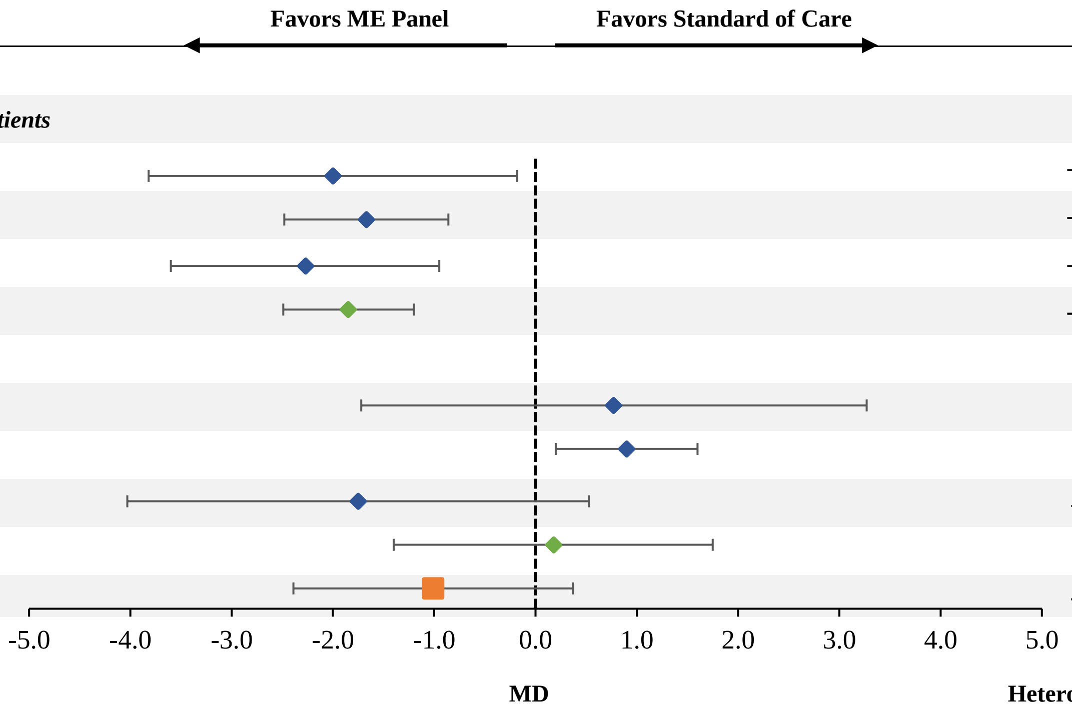

Study	MD (95% CI)	% Weight
Exclusively Pediatric Patients		
Hagen, 2020	-2.00 (-3.82, -0.18)	15.64
McDonald, 2020	-1.67 (-2.48, -0.86)	19.97
O'Brien, 2018	-2.27 (-3.59, -0.94)	17.92
**Overall (n= 3)**	**-1.85 (-2.50, -1.21)**	
Other Patients		
Diaz, 2020	0.77 (-1.73, 3.26)	12.60
Evans, 2020	0.90 (0.20, 1.60)	20.31
Walker, 2021	-1.75 (-4.03, 0.53)	13.54
**Overall (n= 3)**	**0.18 (-1.39, 1.76)**	
**Overall (n= 6)**	**-1.01 (-2.39, 0.37)**	

Heterogeneity, pediatric studies: I^2 = 0.00%
Heterogeneity, other studies: I^2 = 57.91%

CI: confidence interval; MD: mean difference; ME: meningitis and/or encephalitis
